# Comprehensive analysis of mitochondrial dysfunction and necroptosis in intracranial aneurysms from the perspective of predictive, preventative, and personalized  medicine

**DOI:** 10.1007/s10495-023-01865-x

**Published:** 2023-07-06

**Authors:** Bo Chen, Kang Xie, Jianzhong Zhang, Liting Yang, Hongshu Zhou, Liyang Zhang, Renjun Peng

**Affiliations:** 1 Department of Neurosurgery, Xiangya Hospital, Central South University, No. 87 Xiangya Rd., Changsha, 410008 Hunan People’s Republic of China; 2 Hypothalamic-Pituitary Research Center, Xiangya Hospital, Central South University, Changsha, Hunan China; 3 National Clinical Research Center for Geriatric Disorders, Xiangya Hospital, Central South University, Changsha, Hunan China; 4 Department of Surgery, LKS Faculty of Medicine, Queen Mary Hospital, The University of Hong Kong, Hong Kong, China; 5 Department of Neurosurgery, Xiangya Hospital, Central South University (Jiangxi Branch), Nanchang, 330000 Jiangxi China

**Keywords:** Intracranial aneurysm, Mitochondrial dysfunction, Necroptosis, Bioinformatics, 3P medicine

## Abstract

Mitochondrial dysfunction and necroptosis are closely associated, and play vital roles in the medical strategy of multiple cardiovascular diseases. However, their implications in intracranial aneurysms (IAs) remain unclear. In this study, we aimed to explore whether mitochondrial dysfunction and necroptosis could be identified as valuable starting points for predictive, preventive, and personalized medicine for IAs. The transcriptional profiles of 75 IAs and 37 control samples were collected from the Gene Expression Omnibus (GEO) database. Differentially expressed genes (DEGs), weighted gene co-expression network analysis, and least absolute shrinkage and selection operator (LASSO) regression were used to screen key genes. The ssGSEA algorithm was performed to establish phenotype scores. The correlation between mitochondrial dysfunction and necroptosis was evaluated using functional enrichment crossover, phenotype score correlation, immune infiltration, and interaction network construction. The IA diagnostic values of key genes were identified using machine learning. Finally, we performed the single-cell sequencing (scRNA-seq) analysis to explore mitochondrial dysfunction and necroptosis at the cellular level. In total, 42 IA-mitochondrial DEGs and 15 IA-necroptosis DEGs were identified. Screening revealed seven  key genes invovled in mitochondrial dysfunction (KMO, HADH, BAX, AADAT, SDSL, PYCR1, and MAOA) and five genes involved in necroptosis (IL1B, CAMK2G, STAT1, NLRP3, and BAX). Machine learning confirmed the high diagnostic value of these key genes for IA. The IA samples showed  higher expression of mitochondrial dysfunction and necroptosis. Mitochondrial dysfunction and necroptosis exhibited a close association. Furthermore, scRNA-seq indicated that mitochondrial dysfunction and necroptosis were preferentially up-regulated in monocytes/macrophages and vascular smooth muscle cells (VSMCs) within IA lesions. In conclusion, mitochondria-induced necroptosis was involved in IA formation, and was mainly up-regulated in monocytes/macrophages and VSMCs within IA lesions. Mitochondria-induced necroptosis may be a novel potential target for diagnosis, prevention, and treatment of IA.

## Introduction

Intracranial aneurysms (IA) are pathologically localized dilatations and thinning of the cerebral arterial wall, preferentially forming at the bifurcations of the circle of Willis [1]. Based on the shape, IAs can be divided into 3 types: saccular, fusiform, and dissecting. Saccular IAs constitute the majority, occurring in 1–2% of the population [2]. The number of annually detected IAs is continuously increasing with advances in imaging techniques [3]. Subarachnoid hemorrhage caused by IA rupture can be life-threatening with a mortality rate of approximately 35%, resulting  in lasting disabilities and cognitive dysfunction [4]. Current treatments for IAs mainly include two approaches: microsurgical clipping and endovascular management, both of which are invasive, expensive, and exist complications. Therefore, early non-invasive prevention and intervention (e.g. drugs) are particularly vital for IA patients to decrease their health burden and promote health quality. Recently, the concept of predictive, preventive, and personalized medicine (PPPM/3PM) has been introduced for the management of vascular diseases [5] such as arterial stiffness [6], and stroke [7]. From the perspective of PPPM/3PM, we need to identify IA biomarkers for early detection and to understand the molecular mechanisms underlying drug development.

Mitochondria are essential regulators of apoptotic cell death, including necroptosis, ferroptosis, and pyroptosis [8]. Among these, necroptosis is the best-characterized form of regulated necrosis, and is mediated by RIPK3 and MLKL [9]. The mitochondrial-necroptosis axis involves in multiple disease occurrences and developments [10, 11]. It has been highlighted that vascular diseases have potentially benefited from the PPPM/3PM medical strategy targeting mitochondrial dysfunction and necroptosis. Previous research reported that mitochondrial dysfunction could predict the outcome of cardiovascular diseases linked to air pollution [12]. Thus, necroptosis may represent a novel therapeutic target for inhibiting the progression of cardiovascular diseases [13]. Mitochondrial dysfunction of vascular smooth muscle cells (VSMCs) drives the progression of aortic aneurysms [14]. RIPK3-mediated VSMC necroptosis promotes the pathogenesis of aortic aneurysms [15]. Both mitochondrial dysfunction and necroptosis are potential therapeutics for aortic aneurysms [13, 16]. However, studies on mitochondria and necroptosis in IAs are lacking. Identifying the roles of mitochondrial dysfunction and necroptosis in IAs may contribute to the development of  better PPPM/3PM medical strategy for IAs.

Bioinformatics is a crucial component of the transition from traditional medicine to PPPM/3PM [5]. Through bioinformatics, this study aimed to investigate the molecular regulatory mechanism of the mitochondrial-necroptosis axis in IAs, which will contribute to the development of the predictive/diagnostic tools and targeted prevention and therapy from the perspective of PPPM/3PM. In our study, we collected mitochondria-related genes from the MitoCarta3.0 database [17] and necroptosis-related genes from the Kyoto Encyclopedia of Genes and Genomes (KEGG) pathway database. Aiming at mitochondrial dysfunction and necroptosis, the signature genes were selected using weighted gene co-expression network analysis (WGCNA), and phenotype scores were constructed using ssGSEA. We evaluated the association between these two phenotypes using functional enrichment crossover, correlation analysis, immune infiltration, and interaction networks. The key genes of these two phenotypes were validated by machine learning, including Random Forest (RF) and Sequential Minimal Optimization (SMO). Single-cell sequencing (scRNA-seq) was performed to investigate these two phenotypes at the cellular level. Collectively, with the framework of PPPM/3PM, our results elucidate the molecular mechanism of the mitochondrial-necroptosis axis in IAs, and provide a potential target for IA diagnosis, prevention, and treatment.

## Methods

**Fig. 1:**
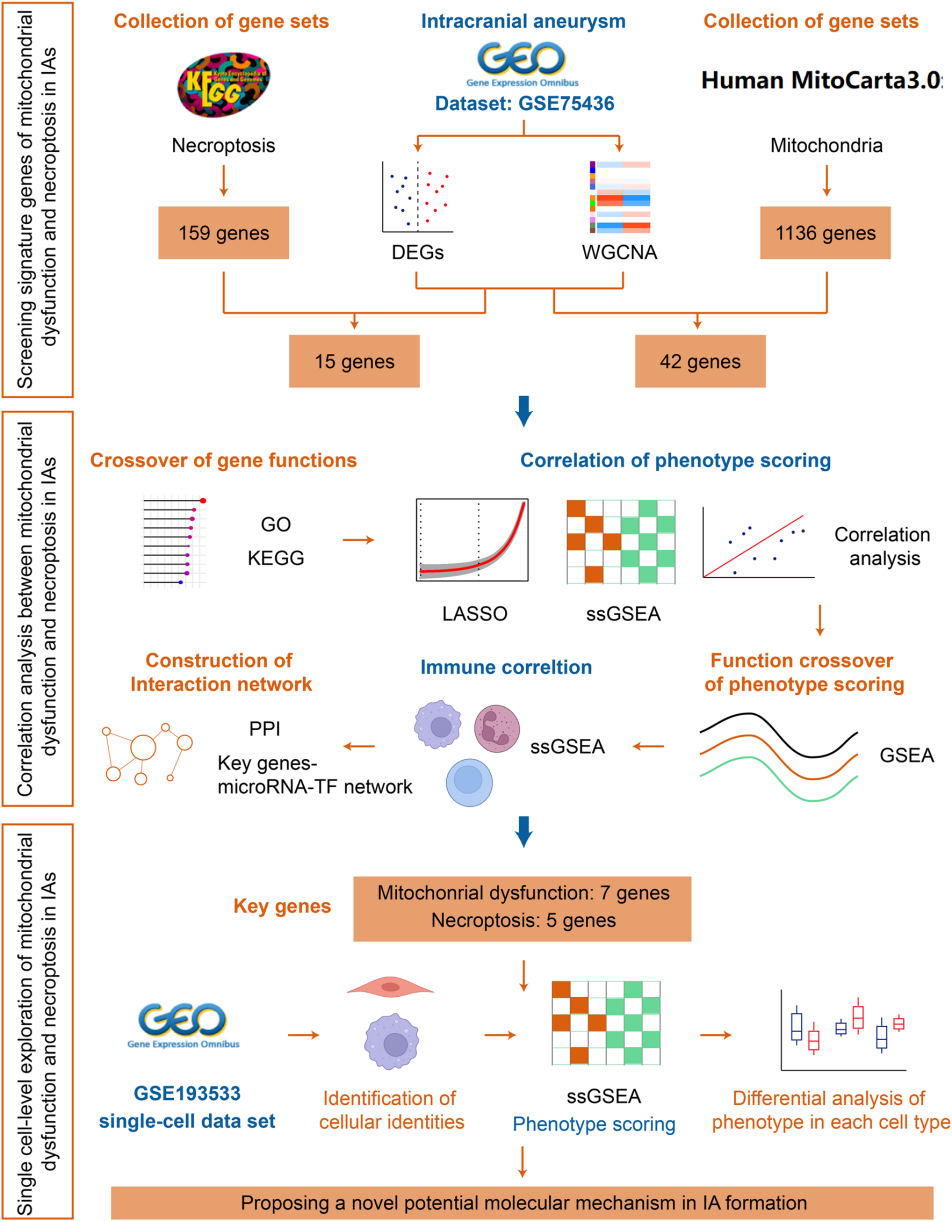
The flow chart of this study

The detailed working flow chart is shown in Fig. 1.

### Data acquisition and processing

IA-related datasets were retrieved from the Gene Expression Omnibus (GEO) database (https://www.ncbi.nlm.nih.gov/geo/), including bulk-tissue mRNA sequence data (GSE75436, GSE15629, and GSE122897) and scRNA-seq data (GSE193533). A total of 75 IA samples and 37 control samples were included, and 4823 cells from the circle of Willis in the sham group and 9986 cells of that in the IA group were collected. The details of these datasets are listed in Table 1. In addition, 1136 human genes encoding proteins that strongly support mitochondrial localization were acquired from the MitoCarta3.0 (https://www.broadinstitute.org/mitocarta). One hundred and fifty-nine necroptosis-related genes were obtained from the KEGG Pathway database (https://www.genome.jp/dbget-bin/www_bget?pathway+hsa04217).

**Table 1:** Descriptions of datasets used in this study

Accession	Platform	Type	Species	Sample
GSE75436	GPL570	mRNA profile	Homo sapiens	IA:control = 15:15
GSE15629	GPL6244	mRNA profile	Homo sapiens	IA:control = 14:5
GSE122897	GPL16791	mRNA profile	Homo sapiens	IA:control = 44:16
GSE193533	GPL30172	scRNA-seq	Mus musculus	IA: sham = 2:1

### Identification of DEGs and IA-related genes

The training set GSE75436 was analyzed using the R package “limma” [18] (P < 0.05 and |log2(FoldChange)| > 1). Differentially expressed genes (DEGs) were identified using heatmaps and volcano plots. WGCNA is a crucial tool in bioinformatic analysis, which has been universally utilized in trait and gene association analysis [19]. In this study, we used the R package “WGCNA” to construct a co-expression network with the gene expression data of GSE75436 as input data and IA/control as trait data. First, the hclust function was used to perform sample clustering to remove outlier samples, and “method = average” was set as the parameter to calculate distance. Second, an appropriate soft threshold was identified to obtain a standard-scale free network. Third, a dynamic shear tree algorithm was employed to segment the modules, and Pearson’s correlation analysis was used to identify the modules related to IA.

### Selection of IA-necroptosis-related DEGs (IA-Necroptosis DEGs) and IA-mitochondria-related DEGs (IA-Mito DEGs)

IA-Mito DEGs were acquired by intersecting the mitochondrial-localization protein-encoding genes, IA-related genes, and DEGs. IA-necroptosis DEGs were identified by intersecting necroptosis-related, and IA-related genes, and DEGs. To decrease the high false discovery rates reported in previous research [20], these genes were further screened using the Wilcox test between the IA and control samples (*P* < 0.05). Potential biological functions were identified by Gene Ontology (GO) and KEGG enrichment.

### Phenotype scoring of mitochondrial dysfunction and necroptosis

IA-Mito DEGs and IA-Necroptosis DEGs were further screened to acquire key genes using the LASSO regression of the R package “glmnet” [21]. Response type was set as binomial and alpha was set as one. The phenotype scores of mitochondrial dysfunction and necroptosis were calculated using the ssGSEA algorithm of the R package “GSVA” [22]. The disparity of phenotype scores between the two groups and the correlation of phenotype scores were analyzed. Furthermore, according to the median values of phenotype scores, the IA and control samples were separately divided into high- and low-groups, and then GSEA analysis [23] was performed for all genes.

### Immune infiltration analysis

Based on a gene set of 28 immune-related cells [24], the immune activity of each sample was evaluated by the ssGSEA algorithm of the R package “GSVA” [22]. The disparity of immune infiltration between the two groups and their correlations were analyzed. Furthermore, the association between the level of immune infiltration and the expression of mitochondrial dysfunction/necroptosis was investigated.

### Construction of interaction networks

The protein-protein interaction (PPI) network between IA-Mito DEGs and IA-necroptosis DEGs was visualized using the STRING website (https://string-db.org) and Cytoscape software (version 3.9.1). The key genes screened from LASSO regression were set as the core of the PPI network, and the confidence level was set to 0.4. To explore the upstream regulation of key genes, we constructed the gene-microRNA-transcription factor (TF) interaction network through the online tool NetworkAnalyst [25]. The parameter of the minimum network was selected.

### Validation of key genes

The expressions of key genes were extracted from the validating sets GSE15629 and GSE122897, and the Wilcoxon test was used to calculate and visualize the expression difference between IA and control samples. We also performed machine learning to evaluate the IA diagnosis value of key genes. To evaluate the classification performance, the 10-fold cross-validation of the RF algorithm was used in dataset GSE75436. The SMO algorithm was employed in the validating sets GSE15629 and GSE122897 with the training set GSE75436.

### scRNA-seq analysis

For single-cell characteristics investigations, we analyzed the scRNA-seq dataset GSE193533, following the “Seurat” standard procedure [26]. Cells with less than 200 genes, more than 7000 counts in total, and more than 20% mitochondria genes were filtered out according to the previous reports [27]. The batch effect among samples was reduced using the R package “harmony” [28]. The top 2000 variably expressed genes were identified using the function “FindVariableFeatures”. The annotation of cell clusters was conducted according to the prior research [29]. Next, the level of mitochondrial dysfunction and necroptosis in each cell was quantified through the ssGSEA algorithm and key genes previously acquired. The expression disparity of these phenotypes was compared between IA and sham groups in each cell cluster.

### Statistical analysis

All statistical analyses were performed using the R software (version 4.1.2). The difference of continuous variables was compared using the Wilcoxon test. The correlation of continuous variables was evaluated by the Pearson correlation. Data were visualized using the R package “ggplot2”. All tests were  two-sided, and *P* < 0.05 was considered statistically significant. The significance level is denoted as follows: NS, not significant; *P < 0.05, **P < 0.01, and ***P < 0.001.

## Result

### Identification of DEGs and IA-Related genes

**Fig. 2:**
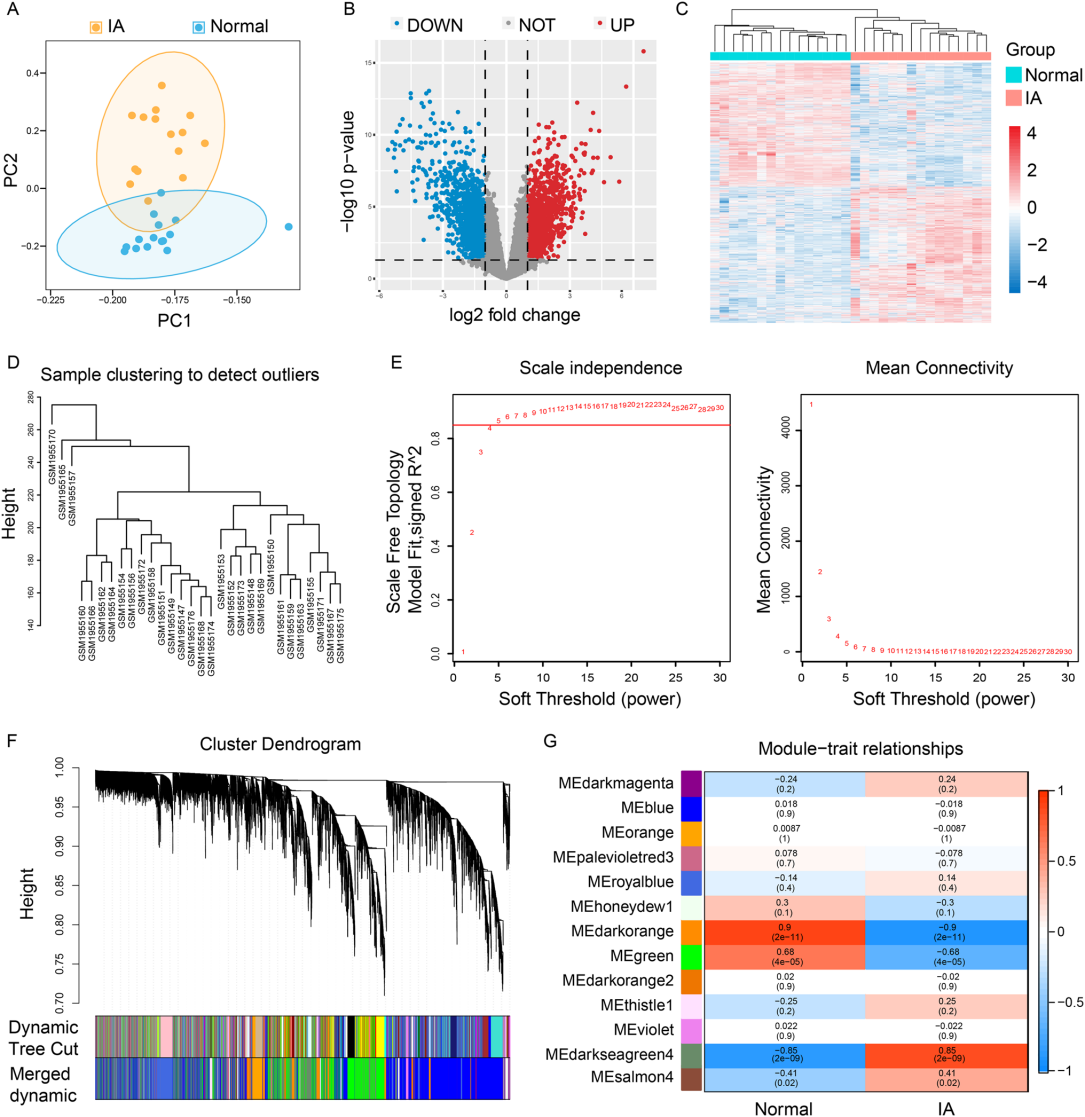
Identification of DEGs and IA-related genes in IA. **A** The PCA results of GSE75436. **B** Volcano plot showing DEGs in the IA and the normal samples. **C** Heat map of DEGs. **D** Sample clustering diagram of WGCNA. **E** Soft-thresholding filtering. **F** Clustering dendrogram of genes. **G** Correlation heatmap of gene modules and clinical features

Based on the expression profile of GSE75436, IA and control groups were dramatically distinguished by PCA (Fig. 2A). Differential expression analysis showed that a total of 2508 DEGs between the IA and control groups, including 1310 genes upregulated and 1198 genes downregulated (Fig. 2B, C). Next, we performed the WGCNA analysis. The sample clustering diagram was shown in Fig. 2D. The correlation coefficient greater than 0.85 (the soft threshold β is 5) was highly correlated and suitable for constructing gene modules (Fig. 2E). The dynamic tree cut algorithm identified 13 gene modules (Fig. 2F). Among them, the darkorange, green, and darkseagreen modules were highly correlated with IAs (|R| > 0.6 and *P *< 0.01) (Fig. 2G). Therefore, the genes in these three modules were regarded as IA-related genes.

### Function crossover of gene between IA-Mito DEGs and IA-necroptosis DEGs

**Fig. 3:**
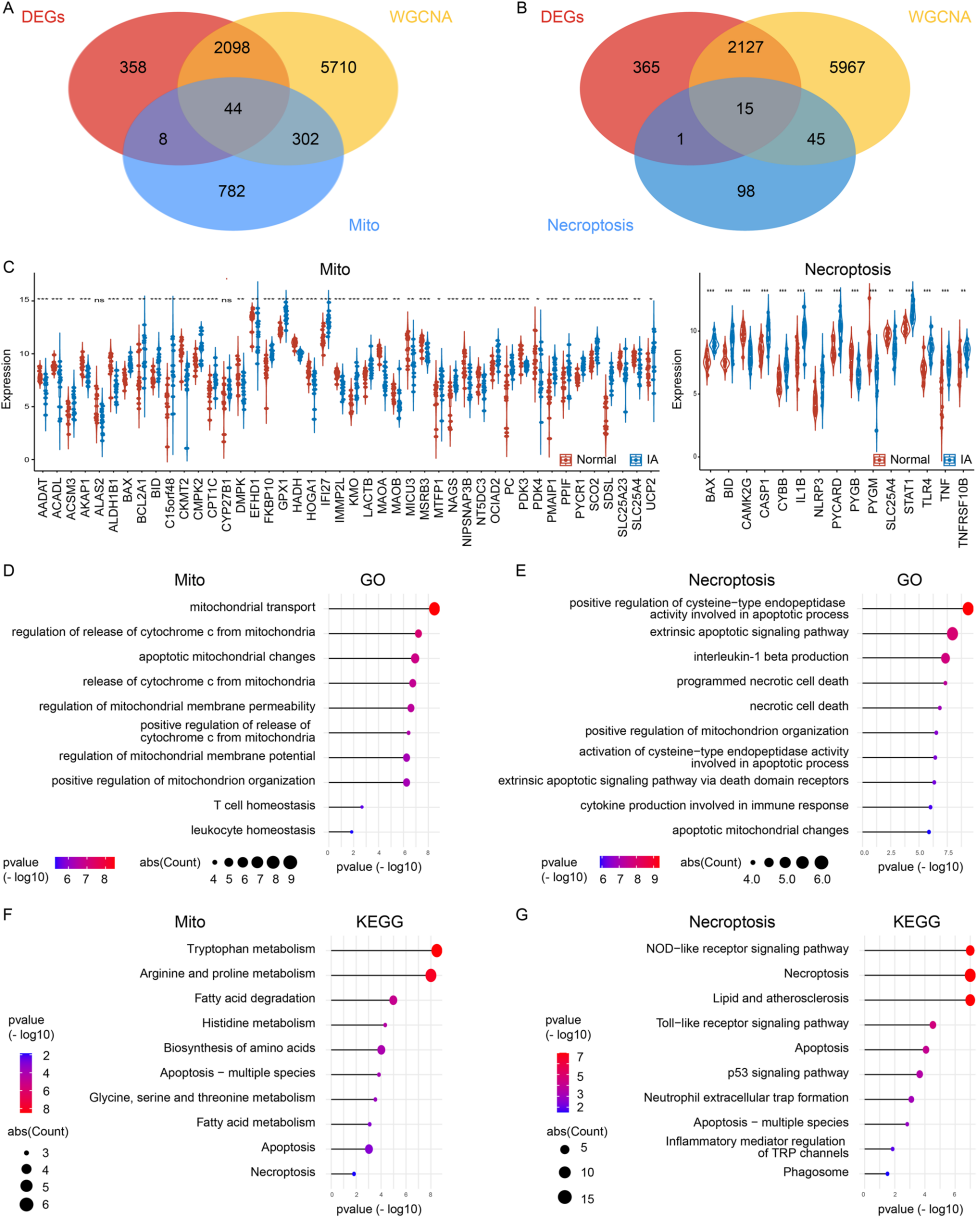
The function crossover of gene between IA Mito DEGs and IA-Necroptosis DEGs. **A** Venn diagram showing the overlap of DEGs, IA-related genes, and mitochondria-related genes. **B** Venn diagram showing the overlap of DEGs, IA-related genes, and Necroptosis-related genes. **C** Expression of 44 IA-Mito DEGs and 15 IA-Necroptosis DEGs in GSE75436. **D** and **E** GO enrichment results of 42 IA-Mito DEGs (**D**) and 15 IA-Necroptosis DEGs (**E**). **F** and **G** KEGG enrichment results of 42 IA-Mito DEGs (**F**) and 15 IA-Necroptosis DEGs (**G**)

We first intersected the DEGs and IA-related genes, and then intersected with mitochondria-related genes and necroptosis-related genes respectively. In total, 44 IA-Mito DEGs and 15 IA-necroptosis DEGs were identified (Fig. 3A, and B). In addition, we screened these genes through the Wilcoxon test between IA and control groups. Two genes ALAS2 and CYP27B1 failed to show significant expression differences and therefore were eliminated, while the remaining 57 genes were used for subsequent analysis (Fig. 3C). Next, the gene functions were explored by GO and KEGG enrichment. In terms of IA-Mito DEGs, the most enriched GO terms were mitochondrial transport, apoptotic mitochondrial changes, T cells homeostasis, leukocyte homeostasis, and the like (Fig. 3D). The most overrepresented KEGG pathways were apoptosis, necroptosis, and so on (Fig. 3F). These results suggest that mitochondrial dysfunction may be related to apoptosis and immunity in IAs. As for IA-Necroptosis DEGs, GO analysis showed that programmed necrotic cell death, apoptotic mitochondrial changes, interleukin-1 beta production, and cytokine production involved in immune response were notable (Fig. 3E). KEGG analysis revealed that necroptosis, neutrophil extracellular trap formation, and inflammatory mediator regulation of TRP channels were enriched (Fig. 3G). These results suggest that necroptosis may be related to mitochondrial dysfunction and immunity in IAs. Overall, the enrichment results showed the gene function of IA-Mito DEGs and IA-necroptosis DEGs existed some level of crossover.

### Function crossover of phenotype scores between mitochondrial dysfunction and necroptosis

**Fig. 4:**
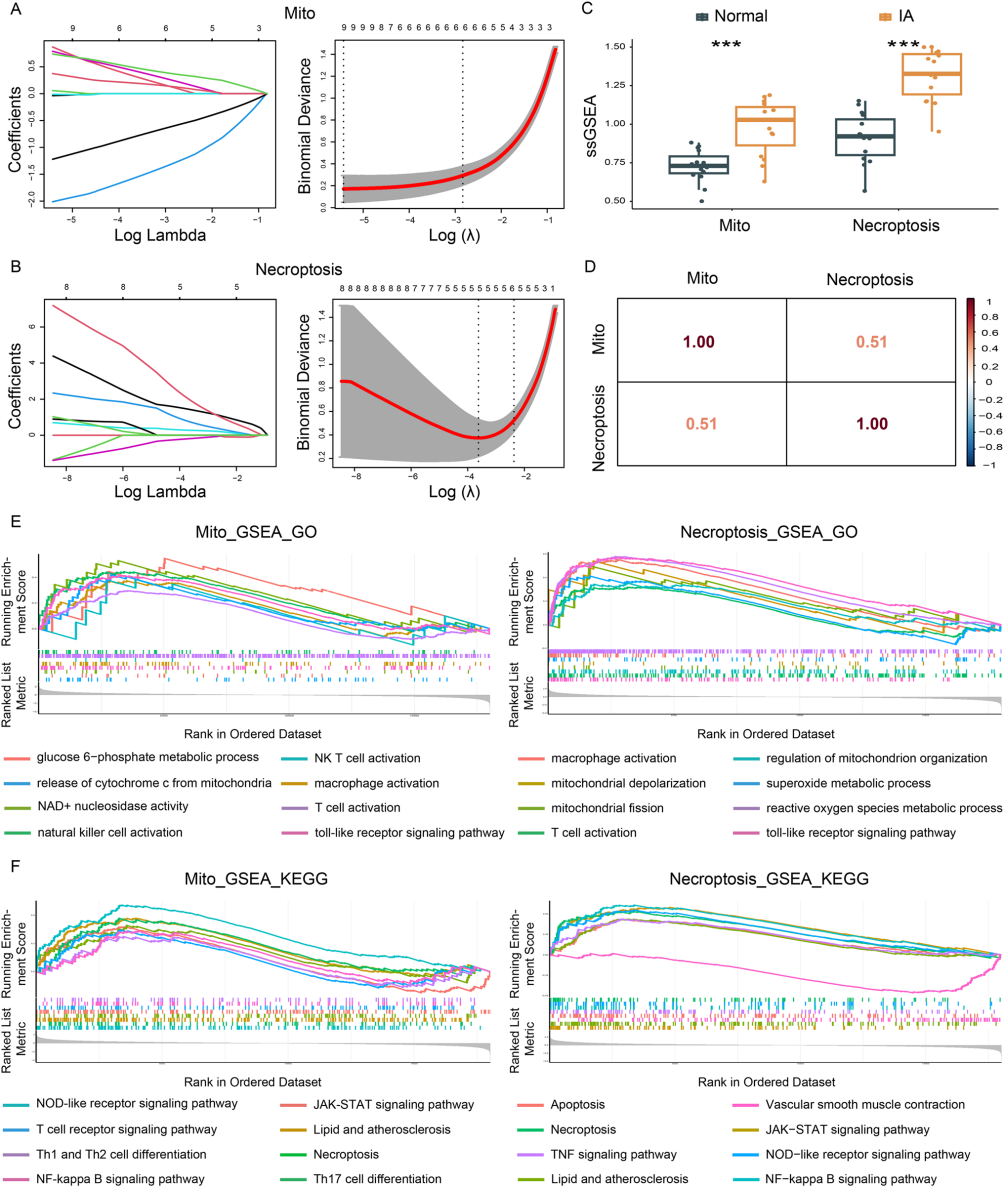
The function crossover of phenotype scores between mitochondrial dysfunction and necroptosis. **A** LASSO regression screened out key genes of mitochondrial dysfunction from the 42 IA-Mito DEGs. **B** LASSO regression screened out key genes of necroptosis from the 15 IA-Mito DEGs. **C** Boxplot of phenotype scores of mitochondrial dysfunction and necroptosis between IA and normal samples. **D** The correlation matrix of phenotype scores of mitochondrial dysfunction and necroptosis. **E** GO enrichment analysis is based on the GSEA algorithm in key genes. **F** KEGG enrichment analysis is based on the GSEA algorithm in key genes

LASSO regression was used to identify key genes with the strongest capacity to predict IA occurrence (Fig. 4A, B). Seven key genes were obtained from 42 IA-Mito DEGs, including AADAT, BAX, HADH, KMO, MAOA, PYCR1, and SDSL. Five key genes were acquired from 15 IA-Necroptosis DEGs, including BAX, CAMK2G, IL1B, NLRP3, and STAT1. These key genes were applied to construct the phenotype scores by the ssGSEA algorithm. As shown in Fig. 4C, IA had a significantly higher level of phenotype scores of mitochondrial dysfunction and necroptosis (P < 0.001). The correlation between these two types of phenotype scores was highly close (R = 0.51) (Fig. 4D). Next, we divided the phenotype scores into high- and low-groups, performed the differential analysis, and conducted the GSEA enrichment analysis for the DEGs. Results showed that both the high mitochondrial dysfunction group and the high necroptosis group had increased levels of mitochondrial pathways (e.g. the release of cytochrome c from mitochondria, mitochondrial depolarization), necroptosis pathways, and immune pathways (e.g. T cell activation) (Fig. 4E, F). These crossover results further suggested the close correlation among mitochondrial dysfunction, necroptosis, and immunocyte infiltration in IAs.

### Correlation of immune infiltration to mitochondrial dysfunction and necroptosis

**Fig. 5:**
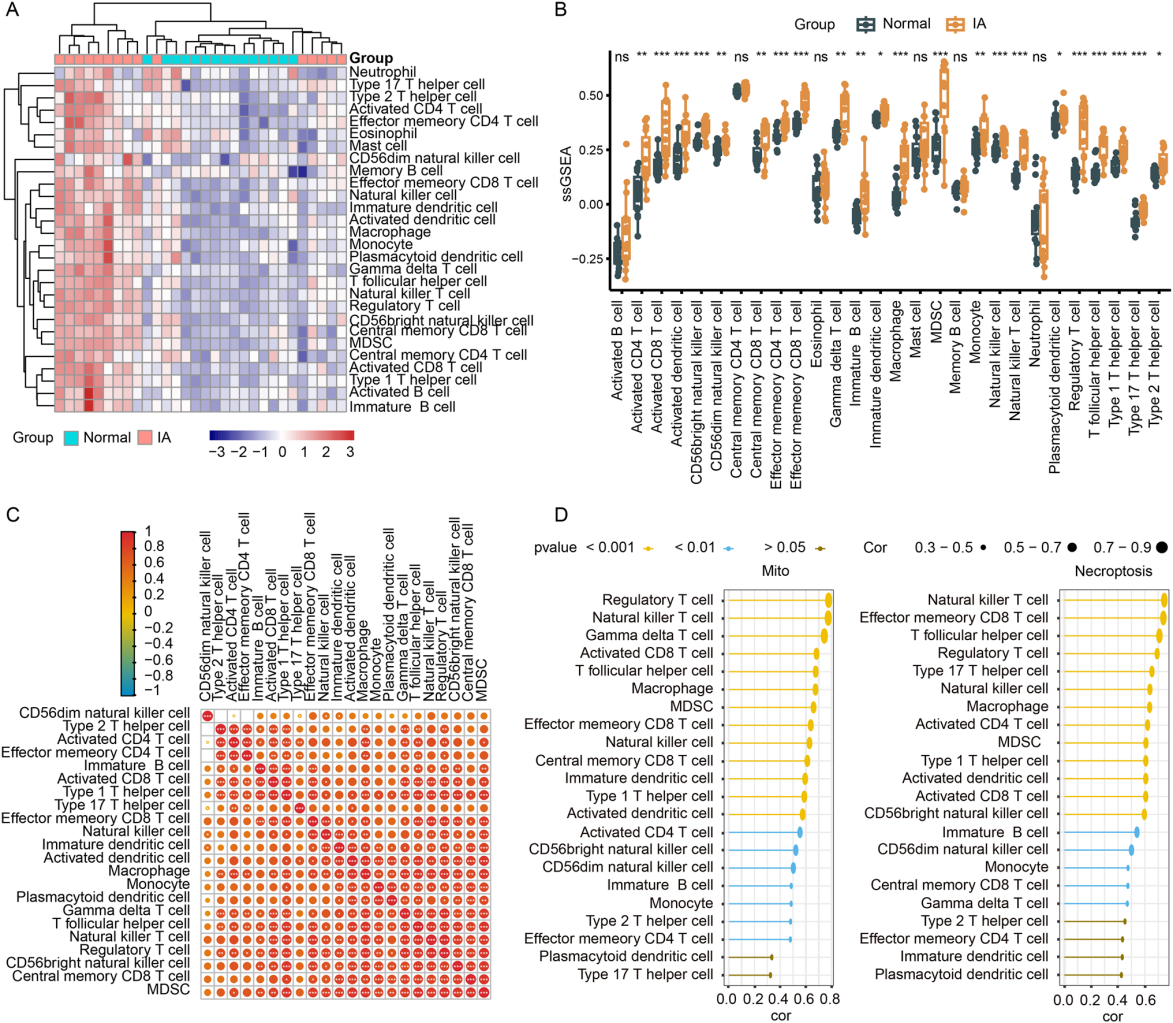
The ssGSEA algorithm for analyzing immunocyte infiltration **A** Heatmap of 28 immune cell types. **B** The boxplot plot of 28 immune gene-sets content. **C** The correlation matrix of immune cells. **D** Correlation diagram for phenotype scores and immunocyte expression

Considering the vital role of immune cells in IA development, we also estimated the level of immune infiltration. Among the 28 immune cells, there were 22 cell types with significant differences between IAs and the controls, and all of them showed higher expression in the IA group (Fig. 5A, B). The expression level of these types of immune cells showed a significantly strong association with each other (Fig. 5C). In addition, the correlation of immune infiltration to mitochondrial dysfunction and necroptosis was evaluated. The phenotype scores of mitochondrial dysfunction and necroptosis were positively correlated to the expression of the 22 types of immune cells. Among them, macrophage was top ranking, with its close relationship to mitochondrial dysfunction (R = 0.675, P < 0.001) and to necroptosis (R = 0.636, P < 0.001) (Fig. 5D).

### Construction of interaction network and validation of key genes

**Fig. 6:**
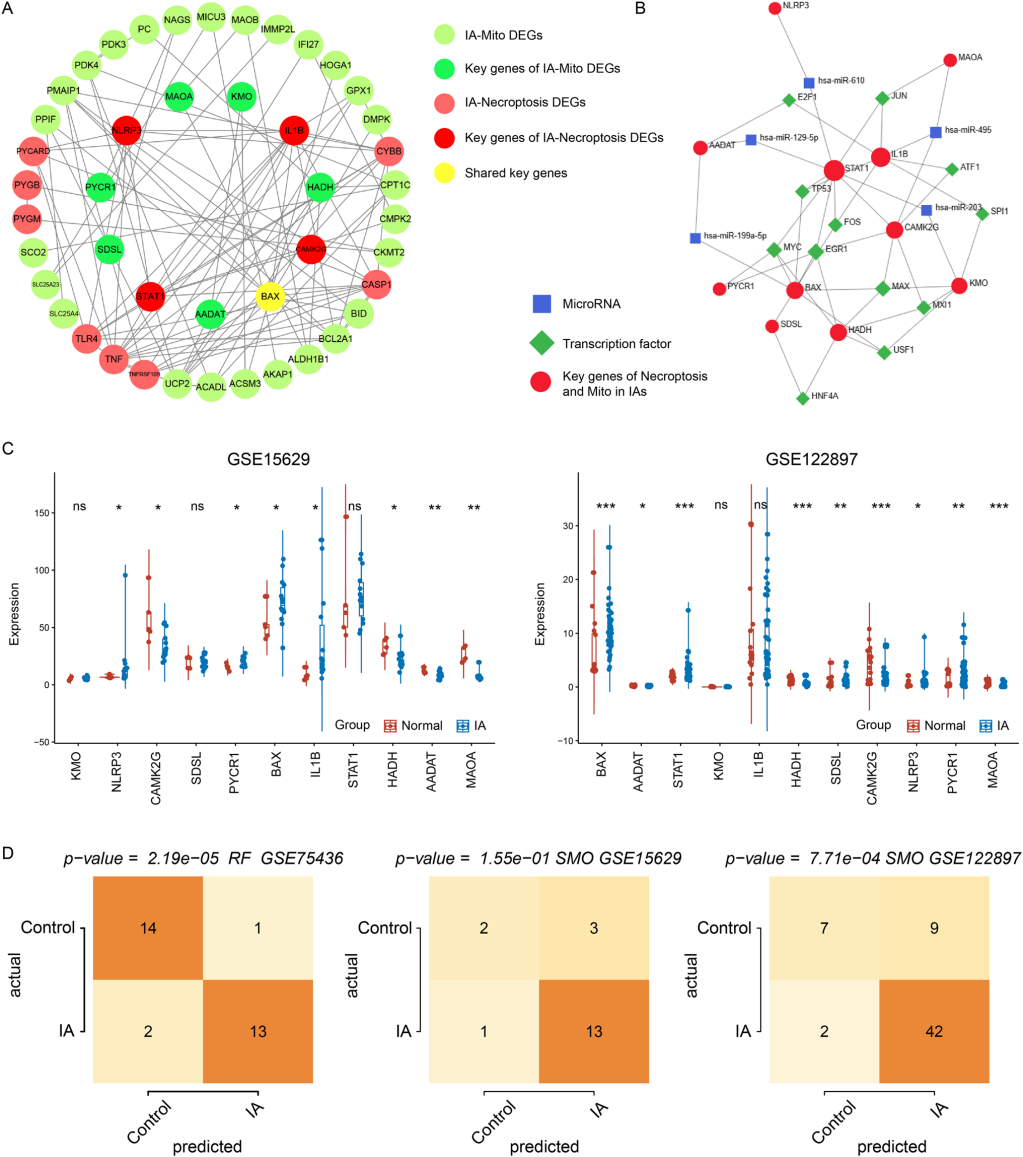
Construction of interaction network and validation of key genes. **A** The construction of PPI network among IA-Mito DEGs, IA-Necroptosis DEGs, and key genes. **B** Prediction of TFs and microRNAs for key genes of mitochondrial dysfunction and necroptosis. **C** Expression of 11 key genes in the validating datasets GSE15629 and GSE122897. **D** Evaluation of IA diagnosis value of key genes by the RF and SMO algorithms

After identifying the correlation between mitochondrial dysfunction and necroptosis, we constructed the PPI network between them. The PPI network included a total of 33 IA-Mito DEGs and 13 IA-Necroptosis DEGs, with the core of 7 key IA-Mito DEGs and 5 key IA-Necroptosis DEGs (Fig. 6A). In addition, the NetworkAnalyst tool was applied to predict the interaction network of key genes, microRNAs, and TFs. JUN, TP53, FOS, EGR1, MYC, MXI1, and USF1 were the common TFs targeting at least 3 key genes. Key genes AADAT and STAT1 had the most predicted microRNAs, including has-miR-129-5p, has-miR-203, and has-miR-199a-5p (Fig. 6B).

Moreover, we used the datasets GSE15629 and GSE122897 to perform the validation of key genes. Most genes exhibited significant expression disparities between IA and control samples. The gene expression trends in the validating set were completely consistent with those in the training set GSE75436 (Fig. 6C). The machine learning showed the high IA diagnosis value of key genes in GSE75436 and GSE122897 (P < 0.05). However, their predictive capability in GSE15629 was unsatisfactory with P > 0.05, which may be influenced by the small samples (Fig. 6D). The predicting IA precisions of key genes were 0.774, 0.902, and 0.811 in GSE15629, GSE75436, and GSE122897. F-measures of the prediction were 0.770, 0.900, and 0.798 in GSE15629, GSE75436, and GSE122897.

### Single-cell level expression of mitochondrial dysfunction and necroptosis

**Fig. 7:**
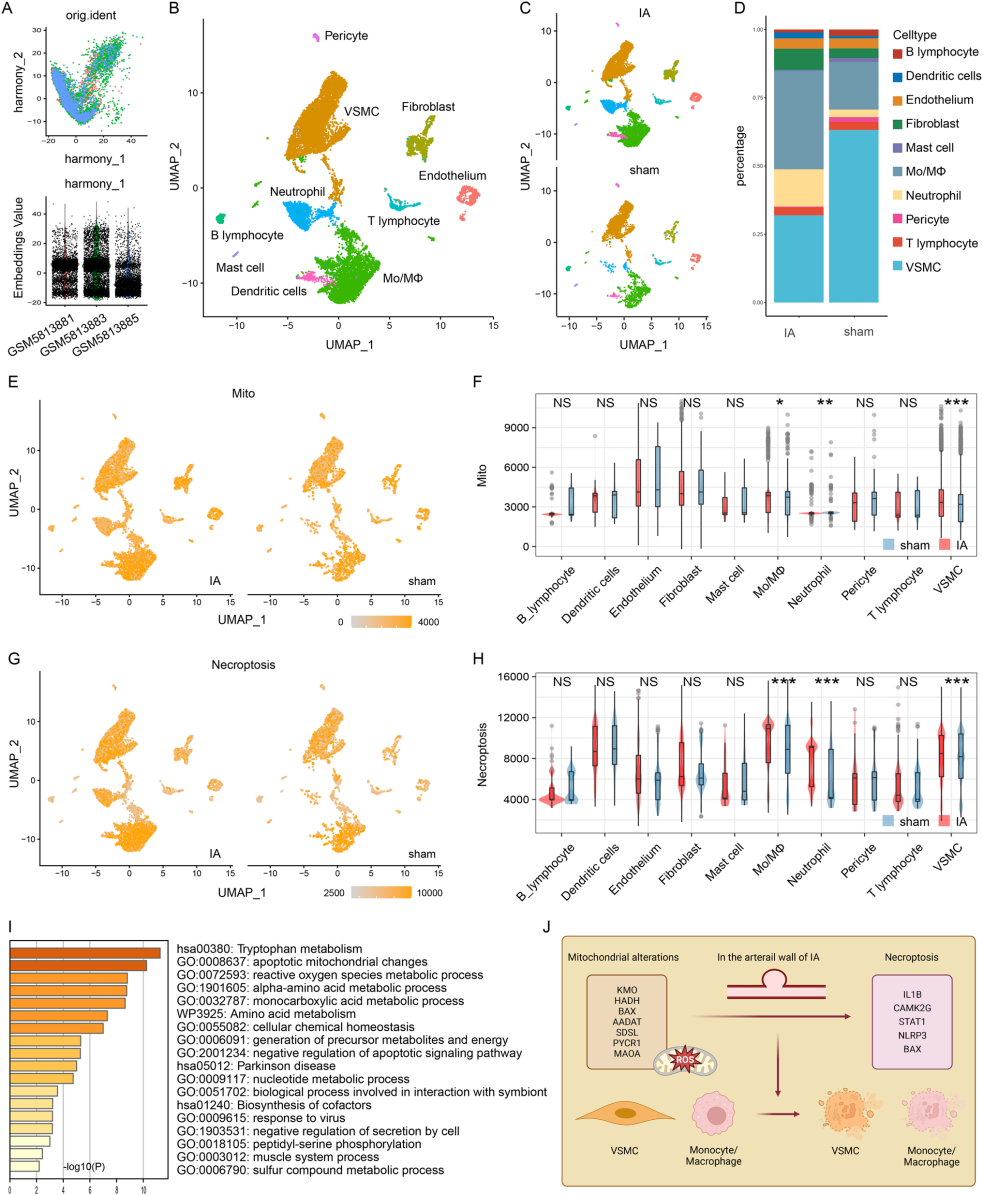
The expression of mitochondrial dysfunction and necroptosis at the cellular level. **A** Integration of multiple sample data using the R package harmony. **B** UMAP plot is colored by different cell types. **C** UMAP plot is colored by different cell types and divided by IA and sham groups. **D** Bar chart showing the percentage of different types of cells between IA and sham groups. **E** UMAP plot is colored by the expression of mitochondrial dysfunction. **F** Bar chart showing the expression disparity of mitochondrial dysfunction between IA and sham groups. **G** UMAP plot is colored by the expression of necroptosis. **H** Bar chart showing the expression disparity of necroptosis between IA and sham groups. **I** GO enrichment results of IA-Mito DEGs in the previously obtained PPI network. **J** Mechanism diagram of mitochondria-induced necroptosis in IAs

To better characterize mitochondrial dysfunction and necroptosis at the single-cell level, we performed scRNA-seq analysis in the circle of Willis of the mouse IA model. After data screening and integration as described in the Methods, we obtained the gene expression profiles of 4823 cells from the sham sample (GSM5813881), and 9986 cells from IA samples (GSM5813883, and GSM5813885) (Fig. 7A). Ten types of cell clusters were annotated and visualized, including VSMC, monocyte/macrophage (Mo/MΦ), and others (Fig. 7B). For ease of comparison, we divided the UMAP figure into 2 pieces: IA and sham (Fig. 7C). The IA samples had dramatically higher proportions of Mo/MΦ and lower percentages of VSMC than sham samples (Fig. 7D).

Next, we quantified the expression of mitochondrial dysfunction and necroptosis, through ssGSEA algorithms and key genes. These two phenotypes were differentially expressed in Mo/MΦ, VSMCs, and neutrophils between IA and sham samples. Compared with sham samples, IA had a significantly higher level of mitochondrial dysfunction and necroptosis in Mo/MΦ and VSMCs. However, the tendency of expression disparity of the two phenotypes was opposite in neutrophils (Fig. 7E–H). Since previous studies have reported a positive correlation between mitochondrial dysfunction and necroptosis [10, 11], we chose to focus on Mo/MΦ and VSMCs, rather than neutrophils. The GO enrichment of the previously obtained PPI network indicated that the reactive oxygen species (ROS) metabolic process was enriched in IA-Mito DEGs (Fig. 7I). Accordingly, combined with the literature review, we proposed the hypothesis that mitochondrial dysfunction (e.g. ROS) may induce the necroptosis of VSMCs and Mo/MΦ in IAs (Fig. 7J).

## Discussion

Mitochondria are important organelles located in the cytoplasm of eukaryotic cells. Besides their roles in cellular metabolism and ATP generation, mitochondria also regulate multiple types of apoptotic cell death, such as necroptosis [8]. Necroptosis is not only caspase-independent but also inhibited by caspase activation, and its morphological features resemble necrosis, including cell swelling, organelle dysfunction, and plasma membrane rupture [30]. Although some studies have elucidated the promoting effects and therapeutic value of mitochondrial dysfunction and necroptosis in aortic aneurysms [14, 15], the relationship between these two phenotypes and IAs remains unclear.

This study focused on the roles of mitochondria dysfunction and necroptosis in IA formation and progression as diagnostic factors and therapeutic targets from the viewpoint of PPPM/3PM. We first used the WGCNA, differential gene analysis, and Wilcoxon test to identify signature genes, including 42 IA-Mito DEGs and 15 IA-necroptosis DEGs. Next, seven mitochondrial key genes and five necroptosis key genes were screened out using the LASSO algorithm. The phenotype scores were quantified through the ssGSEA algorithm. The close relationship between these two phenotypes was identified by function enrichment crossover, correlation analysis, immune infiltration, and interaction network. Subsequently, machine learning verified the high diagnostic value of key genes in IA. The scRNA-seq analysis revealed the concentrated expression of the mitochondrial-necroptosis axis in VSMCs and Mo/MΦ.

Accumulating evidence has demonstrated that mitochondria and necroptosis play crucial roles in aneurysm formation and progression. In our study, IA lesions were associated with  significantly higher mitochondrial dysfunction and necroptosis rates. Key genes of these two phenotypes can well predict IA occurrence. Similarly, previous research found that mice with abnormal mitochondrial calcium uniporter channel complexes were prone to fatal abdominal aortic aneurysms [31]. Sustained increases in mitochondrial dysfunction and oxidative stress have been reported in VSMCs of abdominal aortic aneurysm [32]. RIP3-mediated VSMC necroptosis was actively involved in abdominal aortic aneurysm progression [33]. This preliminary evidence has layed  the foundation for subsequent in-depth analysis.

Mitochondria are essential regulators of cellular necroptosis [9]. The mitochondrial-necroptosis axis is involved in multiple disease occurrences and developments [10, 11]. Chen et al. reported that RIPK1-mediated mitochondrial dysfunction contributed to compression-induced rat pulposus cell necroptosis [34]. Yang et al. found that MiR-7 mediated mitochondrial dysfunction triggered necroptosis in rhabdomyosarcoma [35]. The present study revealed that mitochondrial dysfunction and necroptosis existed the function crossover, high correlation, and network interaction, indicating that the mitochondrial-necroptosis axis may mediate IA pathogenesis. Among the factors inducing mitochondrial dysfunction, ROS (reactive oxygen species) accounts for a significant proportion [36, 37]. Mitochondrial-generated ROS can promote RIPK1 autophosphorylation to initiate necroptosis, ultimately leading to the formation of necrosomes [38, 39]. There has been a strong association between ROS and IA pathogenesis [40]. Cerebral macrophages and VSMCs utilized the main enzymatic sources of ROS to produce O_2_^•−^ and H_2_O_2_ in response to hemodynamic stress, growth factors, and cytokines [41]. Our study indicated that key genes of mitochondrial dysfunction focus on ROS, implying a potentially induced role of ROS in the IA mitochondria-necroptosis axis.

The key genes of the IA mitochondrial-necroptosis axis were identified, including mitochondria genes of seven and necroptosis genes of five. These genes presented a strong interaction in the PPI network. Among them, IL1B[42], BAX [43], STAT1[44], and NLRP3[45] have been considered as potential biomarkers for IA formation and development. BAX, one of the BCL2 protein family members, is an apoptosis regulator. Under stress conditions, BAX would accumulate at the mitochondrial membrane, which results in the release of cytochrome c and triggers cell death [46]. However, the role of BAX in mitochondria-induced necroptosis has not been realved [47]. Moreover, NLRP3 acts as a sensor component of the NLRP3 inflammasome. Previous studies found that NLRP3 inflammasome could be activated by mitochondrial dysfunction [48], and contributes to the necroptosis occurence [49]. Notably, our machine learning analysis revealed that these key genes have a high diagnostic value for IA, which can assist clinicians in identifying early-stage patients.

TFs and microRNAs were involved in the regulation of mitochondrial-necroptosis axis [35]. We drew a microRNA-TF-gene regulatory network on mitochondrial dysfunction and necroptosis in IAs. Seven TFs (JUN, TP53, FOS, MYC, EGR1, MXI1, and USF1) regulated at least three key genes involved in mitochondrial dysfunction and necroptosis. Of them, c-JUN activation is linked to both mitochondrial dysfunction and necroptosis [50, 51]. MYC can increase mitochondrial oxidative phosphorylation and also impede mitophagy-dependent necroptosis [52, 53]. Additionally, JUN [54], TP53[55], FOS [56], SPI1[57], MYC [58], and HNF4A [59] have been proven to participate in IA pathogenesis. In terms of microRNA, we identified hsa-miR-610, hsa-miR-129-5p, hsa-miR-495, hsa-miR-203, and hsa-miR-199a-5p, all of which target the two or more key genes on the mitochondrial dysfunction and necroptosis in IAs. Among these, miR-199a-5p was significantly decreased in IA patients with poor prognosis and vasospasm [53]. MiR-199a-5p was also found to regulate mitochondrial function [60]. Altogether, these TFs and microRNA are promising targets for developing small molecular drugs and novel diagnostic tools in IAs.

To further elucidate the mechanism at the cellular level, we performed the scRNA-seq analysis. Results indicated that both mitochondrial dysfunction and necroptosis expression were significantly higher in Mo/MΦ and VSMC. Macrophages are known to be critical components of immune infiltration and can promote the expression of proteases that disrupt the internal elastic lamina and collagen matrix, leading to the initiation of IA [61]. The mitochondrial-necroptosis axis has been previously reported in macrophages. CIRP has induced mitochondrial DNA fragmentation and regulated macrophage necroptosis [62]. LRRK2 mutations have perturbed mitochondrial homeostasis and reprogrammed necroptosis pathways in macrophages [63]. The close correlation between the mitochondrial-necroptosis axis and immune infiltration in IAs has also been revealed through our functional enrichment and correlation analysis. Additionally, VSMC phenotypic switching from a contractile state to a synthetic state drove the IA formation and rupture [64]. Recent research has reported that mitochondrial DNA damage in VSMCs activated STING signaling and induced cellular necroptosis [65], suggesting the presence of the mitochondria-necroptosis axis in VSMCs. Collectively, mitochondria-induced necroptosis in macrophages and VSMCs may drive IA formation.

This study has some limitations. First, our data came from the GEO database, and the specific clinical data of each sample, such as gender, age, and complications, cannot be obtained, therefore it is not included in our research scope. Second, the sample size obtained from the database was limited. Therefore, studies with a large sample size are needed to thoroughly understand the specific roles of mitochondria and necroptosis in IA. Third, we did not perform in vitro experiments due to the difficulty of obtaining IA samples.

## Conclusions and expert recommendations

In conclusion, our results strongly suggested that mitochondria-induced necroptosis was involved in IA formation. Its key genes had extremely high diagnostic values for IA occurrence. Furthermore, we demonstrated that this mechanism is mainly located in VSMCs and Mo/MΦ within IA tissues. The upregulation of mitochondria-induced necroptosis could be a novel potential target for predictive diagnosis, targeted prevention, and personalized treatment of IAs, which might promote the development of PPPM/3PM in IAs.

### Predictive diagnosis and targeted prevention

From the perspective of PPPM/3PM, mitochondria-induced necroptosis may be a suitable genetic marker for the predictive diagnosis and targeted prevention of IA. The present study screened eleven key biomarker genes of mitochondria-induced necroptosis in IA through integrative bioinformatic approaches (mitochondrial dysfunction: KMO, HADH, BAX, AADAT, SDSL, PYCR1, MAOA; necroptosis: IL1B. CAMK2G, STAT1, NLRP3, BAX). Accordingly, phenotype scores were constructed. Individuals with higher phenotype scores of mitochondria dysfunction and necroptosis are more susceptible to IA. Machine learning further identified their high diagnosis value for IA occurrence. Actually, previous studies had reported that the genes on the mitochondria-induced necroptosis can be a precise method for the diagnosis and prevention of diseases [66]. This is the first time to correlate mitochondria-induced necroptosis with IA. Considering the genetic susceptibility of IAs, we recommend more genetic sequencing studies in terms of mitochondria-induced necroptosis, like exome and intron, to assess the risk of IA occurrence and rupture.

### Personalized medicine

Although growing evidence suggested that both mitochondrial dysfunction and necroptosis are potential therapeutics for aortic aneurysm [13, 16], no study noted the application of these two phenotypes in IA treatment. Therefore, we recommend focusing on mitochondria-induced necroptosis in IAs. These eleven key biomarker genes can help researchers to design and develop novel small molecular drugs, which may inhibit IA formation and progression. The key gene-miRNA-TF regulatory network would also provide a unique benefit to developing new approaches in IA treatment. Of note, since the mitochondria-induced necroptosis is mainly located in Mo/MΦ, we strongly recommend developing customized immunotherapy in IAs from the perspective of PPPM/3PM.

## Data Availability

The datasets generated and/or analyzed during the current study are available in the GEO database, including the datasets GSE75436 (https://www.ncbi.nlm.nih.gov/geo/query/acc.cgi?acc=GSE75436), GSE15629 (https://www.ncbi.nlm.nih.gov/geo/query/acc.cgi?acc=GSE15629), GSE122897 (https://www.ncbi.nlm.nih.gov/geo/query/acc.cgi?acc=GSE122897), and GSE193533 (https://www.ncbi.nlm.nih.gov/geo/query/acc.cgi?acc=GSE193533).

## Abbreviations

DEG: Differentially expressed gene
GEO: Gene Expression Omnibus
GO: Gene Ontology
IA-Mito DEGs: IA-mitochondria-related DEGs
IA-Necroptosis DEGs: IA-Necroptosis-related DEGs
IA: Intracranial aneurysm
KEGG: Kyoto Encyclopedia of Genes and Genomes
MMP: Matrix metalloproteinases
Mo/MΦ: Monocyte/macrophage
PPPM/3PM: Predictive, preventive, and personalized medicine
PPI: Protein-protein interaction
RF: Random Forest
ROS: Reactive oxygen species
SMO: Sequential Minimal Optimization
scRNA-seq: Single-cell RNA sequencing
TF: Transcription factor
VSMC: Vascular smooth muscle cell
WCGNA: Weighted gene co-expression network analysis

## Code availability

All software applications used are included in this article.
